# Economic evaluation of orphan drug Lutetium-Octreotate vs. Octreotide long-acting release for patients with an advanced midgut neuroendocrine tumour in the Netherlands

**DOI:** 10.1007/s10198-021-01303-2

**Published:** 2021-04-07

**Authors:** Marije E. Hagendijk, Simon van der Schans, Cornelis Boersma, Maarten J. Postma, Simon van der Pol

**Affiliations:** 1 Departments of Economics, Econometrics and Finance, Faculty of Economics and Business, University of Groningen, Groningen, The Netherlands; 2 Coronel Institute of Occupational Health and Research Center for Insurance Medicine, Amsterdam UMC, University of Amsterdam, Amsterdam Public Health Research Institute, Amsterdam, The Netherlands; 3 Department of Health Sciences, University of Groningen, University Medical Center Groningen, Groningen, The Netherlands; 4 Fair Medicine Foundation, Amsterdam, The Netherlands; 5 Faculty of Management Sciences, Open University, Heerlen, The Netherlands; 6 Institute of Science in Healthy Aging and Healthcare (SHARE), University of Groningen, University Medical Center Groningen, Groningen, The Netherlands

**Keywords:** Economic evaluation, ^177^Lu-DOTATATE, Lutetium-Octreotate, Neuroendocrine tumour, Octreotide long-acting release

## Abstract

**Objectives:**

Multiple studies showed positive effects of Lutetium-Octreotate (LO) treatment in neuroendocrine tumours. LO has been used in the Netherlands since the 1980s and recently received the orphan status shortly after the acquisition by Novartis. Since then, the official list price has increased sixfold. From a value-based pricing perspective, we analysed the impact of the increase in price on the incremental cost-effectiveness ratio (ICER) of LO treatment compared to optimal best supportive care, a high dose of Octreotide long-acting release (O-LAR), using the clinical data of the NETTER-1 trial.

**Methods:**

A Markov model was developed to evaluate the costs per quality-adjusted life-year (QALY) for LO treatment compared to O-LAR from the healthcare perspective. A scenario analysis was conducted to compare the cost-effectiveness with the initial and increased price level of the LO-treatment.

**Results:**

At the increased price level, the cost-effectiveness analysis rendered a deterministic ICER of €53,500 per QALY, while at the initial pricing, the ICER was €19,000 per QALY. The probabilistic sensitivity analysis (PSA) showed that LO had a high probability of being cost-effective at both the increased and initial price level, considering a cost-effectiveness threshold of €80,000.

**Conclusions:**

Even at the increased price level, LO treatment can still be considered cost-effective using the applicable Dutch willingness-to-pay threshold of 80,000 euro per QALY. Considering the public scrutiny in relation to this price increase, these outcomes raise the question whether traditional cost-effectiveness methods are sufficient in fully capturing the societal acceptance of prices of new medicines.

**Supplementary Information:**

The online version contains supplementary material available at 10.1007/s10198-021-01303-2.

## Introduction

Pricing of medicines is a growing concern in most healthcare systems with accessibility and affordability increasingly in the forefront: high pricing of medicines is increasingly considered to be unacceptable. Medicine prices are a consequence of the principles the pharmaceutic industry applies and rules and regulations for reimbursement. The most common pricing principle is value-based pricing, i.e., a pricing approach which sets prices primarily aligned to the value of a product rather than according to the cost of the development of the product [1]. Value in healthcare consists of different elements, that are either core elements or common elements that are inconsistently used [2]. In addition to this, novel elements of value, like value of hope or scientific spillover, might be introduced in the future [2]. Consequently, innovation can be rewarded highly if medical need and potential cost savings and added value are estimated to be relevant. While value-based pricing clearly includes patient-reported outcome measures (PROMs) and potential cost offsets [3], it does not include other societal values, such as affordability, accessibility, opportunity costs [4] of medicine, and the need for transparency in pricing methods and structure [1]. A broader approach would be needed to incorporate such additional types of value in the pricing process and reimbursement decision. As a recent example where it has often been felt that additional considerations would come in, in this paper, we address the concept of value-based pricing and societal acceptance of Lutetium-Octreotate (LO) pricing in the Netherlands.

In the 1990s, a new type of treatment for neuroendocrine tumours, labelled peptide receptor radionuclide therapy, was developed. Neuroendocrine tumours are slow-growing tumours located in hormone-producing cells of the body, such as the pancreas and the midgut [5]. This type of tumour is rare; cumulatively from 1990 to 2010, the recorded incidence was 47,800 patients, in the Netherlands [5–7]. The therapy targets and binds the somatostatin receptors on the surface of the hormone-producing cells, after which its radioactive character provides local radiotherapy to the tumour sites. At this moment, the only form of peptide receptor radionuclide therapy approved in Europe is LO treatment (Novartis International AG, Basel, Switzerland), also called ^177^Lu-DOTATATE*,* a combination of Lutetium-177 (IDB Holland BV, Baarle-Naussau, Netherlands) with the amino acid peptide DOTA-octreotate. LO was developed by researchers since 1985 and introduced as peptide receptor radionuclide therapy treatment in 1992 in the Erasmus Hospital in Rotterdam, The Netherlands (Biosynthema Inc.).

In 2011, the French pharmaceutical company Advanced Accelerator Applications (AAA) became the owner of LO. AAA financed the research and development costs for a randomized controlled phase III trial, executed between 2012 and 2015 and published in 2017 as the NETTER-1 study [8]. Together with earlier publications of case reports by Biosynthema Inc [9–11], this additional phase III trial provided enough evidence for the European Medicines Agency to give LO treatment market authorisation in 2018. In the same year, Novartis Pharmaceuticals acquired LO, and a few days after the acquisition, LO treatment obtained the orphan drug status [12]. Consecutively, LO treatment’s price was raised from €16,000 pre-acquisition (initial) to €90,000 post-acquisition (increased) per treatment, for four injections [13].

The Dutch national cancer organisation and Dutch healthcare insurers suggested abuse of the monopoly position for LO [13, 14]. In response, it was claimed that the medicine is cost-effective in other European countries with the new price [14]. To our knowledge, only one study concluded that it was unlikely that LO would be cost-effective for England and Wales [15]; for other countries including the Netherlands, no cost-effectiveness analyses exist in the published literature.

The purpose of this paper is to conduct an economic evaluation of LO for the Netherlands, based on the Dutch guidelines for pharmacoeconomic research [16], in the perspective of added value, value-based pricing, and acceptance of pricing; using the clinical data from the NETTER-1 study [8] and considering both the initial and increased prices of LO. Notably, for diseases with high severity, the Netherlands applies a Willingness-To-Pay (WTP) threshold of €80,000 per Quality-Adjusted Life Year (QALY) for the value-based perspective of the Dutch Care Institute [17].

## Methods

For the evaluation of the cost-effectiveness of LO compared to best supportive care, i.e., treatment with high-dose Octreotide long-acting release (O-LAR), we used data from the NETTER-1 trial [8]. In line with the trial, LO treatment was assumed to consist of four injections, one every 8–10 weeks. Additionally, patients received O-LAR 30 mg injections 24 h after each LO injection and monthly after the completion of all four injections, as maintenance therapy for symptom control. The control group received injections of O-LAR 60 mg every 28 days. Both treatments were stopped when the patients showed progressive disease measured by a computed tomography (CT) scan or magnetic resonance imaging (MRI).

### Patient population

The data included in this economic evaluation were derived from the patient population studied in the NETTER-1 study [8]. This patient population (*N* = 229) was randomly divided into the two study groups: LO treatment plus standard-dose O-LAR (intervention arm, *N* = 116) and high-dose O-LAR (control arm, *N* = 113). The LO group had an average age of 63 (SD ± 9) years with 54% being male and the high-dose O-LAR-group 64 (SD ± 10) years and 47% being male. The most common primary tumour site in both groups was the ileum (74 and 73%, respectively) and the most common site of metastasis in both groups was the liver (84 and 83%, respectively).

### Model design and time horizon

**Fig. 1:**
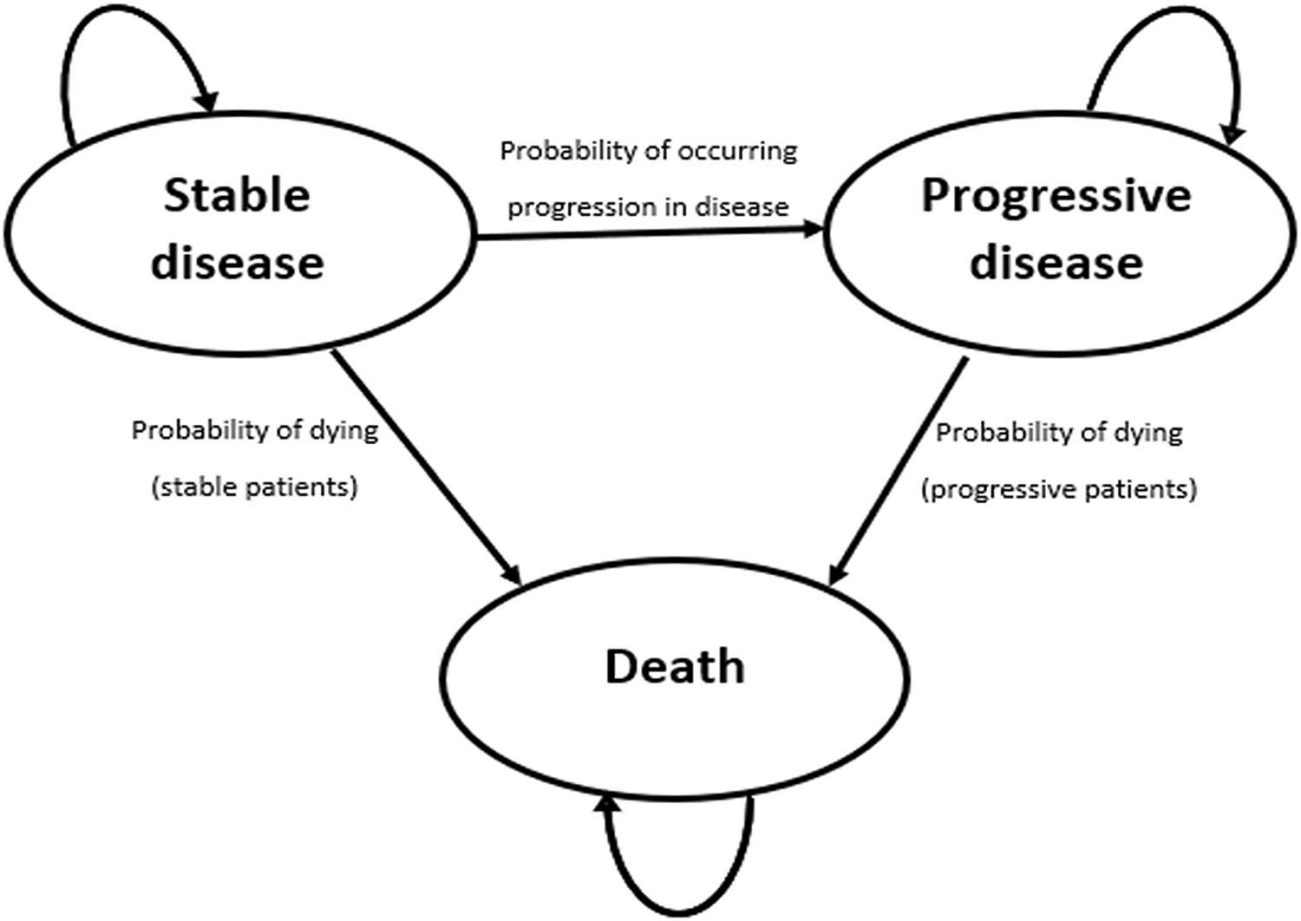
The Markov model; the circles indicate health states and the arrows indicate transitions between the health states

We developed a Markov model with three health states (Fig. 1):

- Stable disease (SD), with no present growth in tumour size or even a reduction in the tumour size;
- Progressive disease (PD), with growth in tumour size being visible; and
- Deceased patients.

Within this Markov model, a cohort of 1.000 63-year-old patients with advanced neuroendocrine tumour was modeled. We used a cycle duration of 28 days and considered a lifetime horizon. For each cycle, the utility values, Life Years Gained (LYG) and total costs of both arms, were followed, and subsequently, differences in totals were inserted in the Incremental Cost-Effectiveness Ratio (ICER). The Markov model was designed and build within Microsoft Excel^®^ 2016 (Microsoft Corp, Redmond, WA).

### Transition probabilities

The transition probabilities were derived from the progression-free and overall survival curves from the NETTER-1 study and its first update [8, 18] using the control arms as the reference. We did not have access to the individual patient data of the NETTER-1 study and the data points from these curves were distracted from the papers [8, 18] using Web Plot Digitizer [19]. The data from the Kaplan–Meier curves were reconstructed using the method explained by Guyot et al. [20] and the best-fitting parametric curves were selected by plotting them using R 3.6.3 [21] and the flexsurv package [22]. For the overall survival, a gamma function was selected and for the progression-free survival, an exponential function. See supplementary appendix 1 for a list of functions considered and the figures of the functions used for this analysis.

In the original publication and the first update [8, 18], hazard ratios for the progression-free and overall survival of the LO group compared to the control group were given. A reported hazard ratio for survival in the health state SD as compared to PD [23] was used to differentiate the survival rates for both health states. These hazard ratios were applied to the transition probabilities in each time cycle (Table 1).

**Table 1:** Parameters used in the model; survival rates, transition probabilities and utility values

Effects	HR (± 95% CI)	Source
Progression-free survival^a^	0.21 (0.14–0.33)	[8]
Overall survival^a^	0.54 (not given)^+^	[18]
Overall survival SD vs. overall survival PD^b^	0.44 (0.24–0.79)	[23]

Transition	Patient group	Probability (± 95% CI)
SD-PD^c^	High dose O-LAR	0.0854
	LO + O-LAR	0.0183 (0.0119**–**0.0283)
SD-deceased^d^	High dose O-LAR	0.0095
	LO + O-LAR	0.0073 (0.0059**–**0.0088)^e^
PD-deceased^d^	High dose O-LAR	0.0216
	LO + O-LAR	0.0116 (0.0093**–**0.0139)^e^

Utility [19]	Mean value (min–max)	Distribution
SD without/grade I or II AE	0.771 (0.509**–**0.818)	Beta
SD with grade III/IV AE		
High-dose O-LAR	0.666 (0.619**–**0.709)	Beta
LO + O-LAR	0.687 (0.642**–**0.730)	Beta
PD	0.612 (0.564**–**0.659)	Beta
Deceased	0.000 (0.000**–**0.000)	None

*AE*  adverse events, *CI* confidence interval, *HR*  hazard ratio, *LO* Lutetium-Octreotate, *O-LAR* Octreotide long-acting release, *PD* progressive disease, *SD* stable disease

^a^Effect of LO treatment compared to the high dose of O-LAR treatment

^b^Effect of O-LAR treatment overall survival SD compared to O-LAR treatment overall survival PD

^c^Exponential distribution over time

^d^Gamma distribution over time

^e^Assumed 80%-120% interval

The probabilities of dying due to other causes than a neuroendocrine tumour were modeled according to the age-dependent mortality rates of the general population as taken from the Dutch Statistics of public health [24]. These mortality rates were used exclusively to model the mortality probabilities from the time cycle where this probability was higher than the mortality reported in the NETTER-1 study [8].

### Utilities

Unfortunately, no utilities were published for the Dutch population. The utilities for the different health states were derived from a time trade-off study from the United States [25], as they provided a clear distinction between the health states used in our model as well as reflecting assessments being population-based for neuroendocrine tumours. Utilities for SD without/with low-grade adverse event, SD combined with one kind of severe or life-threatening adverse event and PD, were derived from this study and the same for both study arms. The utility values of the SD including grade III and IV adverse events for both treatment arms were calculated based on the percentages experiencing a different grade III and IV adverse event [8, 25, 26]. Note that not for all adverse events captured with the NETTER-1 study [8], a utility value was presented [25]. The utility values included in our model were based on the adverse events for which a utility value was measured [25], which can be found in Table 1. In the probabilistic analysis, utility values were assumed to follow a beta distribution [20].

### Costs

The economic evaluation was executed from the healthcare perspective. As the researchers did not have access to the individual patient data of the NETTER-1 study [8], indirect non-medical costs such as productivity losses or volunteer time were not included in the analysis. Medicine costs, costs to manage adverse events, medical resource utilization costs, and indirect medical costs in life-years gained were considered.

Medicine costs were taken from the official price list as published by the Dutch National Healthcare Institute [27, 28]. Medical resource use costs were derived from the Dutch healthcare cost manual [29]. When patients shift to the PD health state, the LO and high-dose O-LAR treatment costs reduce to zero, as reported in the NETTER-1 study [8]. Outpatient follow-up visits for blood testing, with a frequency of every 6 months, reflect the only remaining direct health care costs. The grade III and IV adverse event’s costs were based on the data of the Safety Assessments from the supplementary appendix of NETTER-1 [8].

Annual, indirect unrelated medical costs were derived from the Dutch PAID 1.1 tool [30]. This tool provides the mean medical costs which are not related to the direct costs of the disease itself based on the incidence of other diseases for a particular age and gender in life-years gained. The costs of pancreas cancer were selected as proxy for the direct medical costs of neuroendocrine tumours to be excluded from the indirect medical costs to avoid double counting. According to the PAID 1.1 tool, indirect medical costs were divided into end-of-life costs and costs in other stages.

A detailed overview of the included costs is displayed in Supplementary appendix 2. All costs in the paper were reported in 2019 euros, when necessary, cost data were converted to 2019 using the consumer price index [31]. The ICER values were rounded to the nearest hundreds of euros.

### Discounting

Annual discount rates for costs of 4% (0.3% per monthly cycle) and for health of 1.5% (0.11% per cycle) per year were taken into account according to the Dutch pharmacoeconomic guidelines [16]. A sensitivity analysis was performed with equal discounting for both costs and QALYs at 0 and 4%.

### Willingness to pay

The Dutch healthcare system makes use of varying willingness-to-pay (WTP) thresholds, depending on the burden of disease. For cancer treatments, the highest threshold level is applicable, due to the high burden of disease [17]. A calculation of the burden of disease with the modeled outputs [32], showed a 99% probability that the applicable willingness to pay is €80,000 per QALY (Supplementary appendix 3).

### Sensitivity analysis

Since we focus on the increase in costs of the LO treatment, both the initial and increased price levels were considered in our analysis. A PSA was executed within Microsoft Excel^®^ 2016 by a Markov Chain Monte Carlo simulation sampling 10,000 values. For the input parameters without confidence intervals provided in the literature, such as the hazard ratio of the overall survival, a lower and higher bound of 80 and 120% of the deterministic means were assumed. The outcomes of the executed PSA were translated into cost-effectiveness acceptability curves (CEACs) [33].

Effects of implementing the lower and higher bounds of included parameters, as utility values, discount values, costs, hazard ratios, and probabilities, were analysed using a deterministic sensitivity analysis and represented in a Tornado Diagram [34]. This Tornado Diagram also incorporated the effects when excluding the indirect medical costs, and when excluding the follow-up for the progression-free survival after 25 months, since the follow-up was extrapolated after the 25th month for all cycles. For the overall survival, the inclusion of general population survival rates was analysed. An extrapolation of the survival from the NETTER-1 study for the full-time horizon was added as a sensitivity analysis to study the effect of using the age-dependent mortality probability if this was higher than the probability measured in the NETTER-1 study [8].

## Results

### Deterministic analysis

The ICER at the increased list price of LO is €53,500 per QALY, while the initial price of LO renders an ICER of €19,000 per QALY. See Table 2 for the results of the deterministic cost-effectiveness analysis.

**Table 2:** Results of deterministic cost-effectiveness and life-years-gained analysis (discounted)

Treatment	Costs^a^ (€)	QALY^a^	LYG^a^	ICER (€ per QALY)	ICER (€ per LYG)
High dose O-LAR	70,491	1.79	2.68		
LO initial price	105,258	3.61	5.09	19,000	14,500
increased price	168,216	3.61	5.09	53,500	40,600

^a^Total from the start of treatment until end of lifelong (> 99% in absorbing state), mean age of both groups was 63.5y. Costs and ICER values rounded to hundreds

*ICER*  incremental cost-effectiveness ratio, *LYG* life-year gained, *O-LAR*  Octreotide long-acting release, *QALY*  quality-adjusted life-years

### CE plane and CEAC

**Fig. 2:**
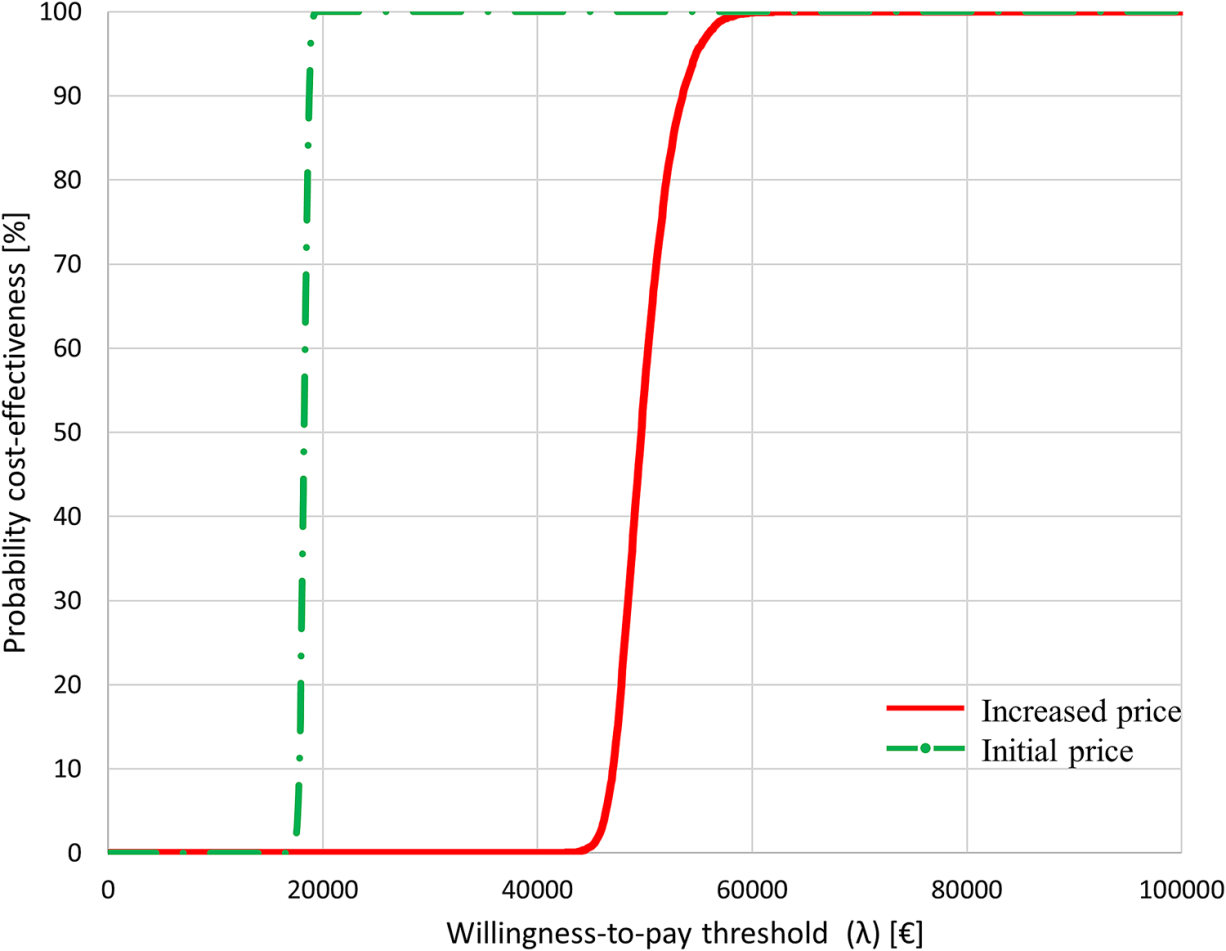
Cost-effectiveness acceptability curve

Figure 2 gives the CEACs at both price levels. At a WTP threshold of €50,000 per QALY, the estimated probability of LO being cost-effective is 55% at the increased price and 100% at the initial price. For the WTP threshold of €80,000 per QALY, LO is cost-effective at both price levels. In supplementary appendix 4, the CE plane is given, showing the distribution of the Monte Carlo analysis for both price levels.

### Deterministic sensitivity analysis

**Fig. 3:**
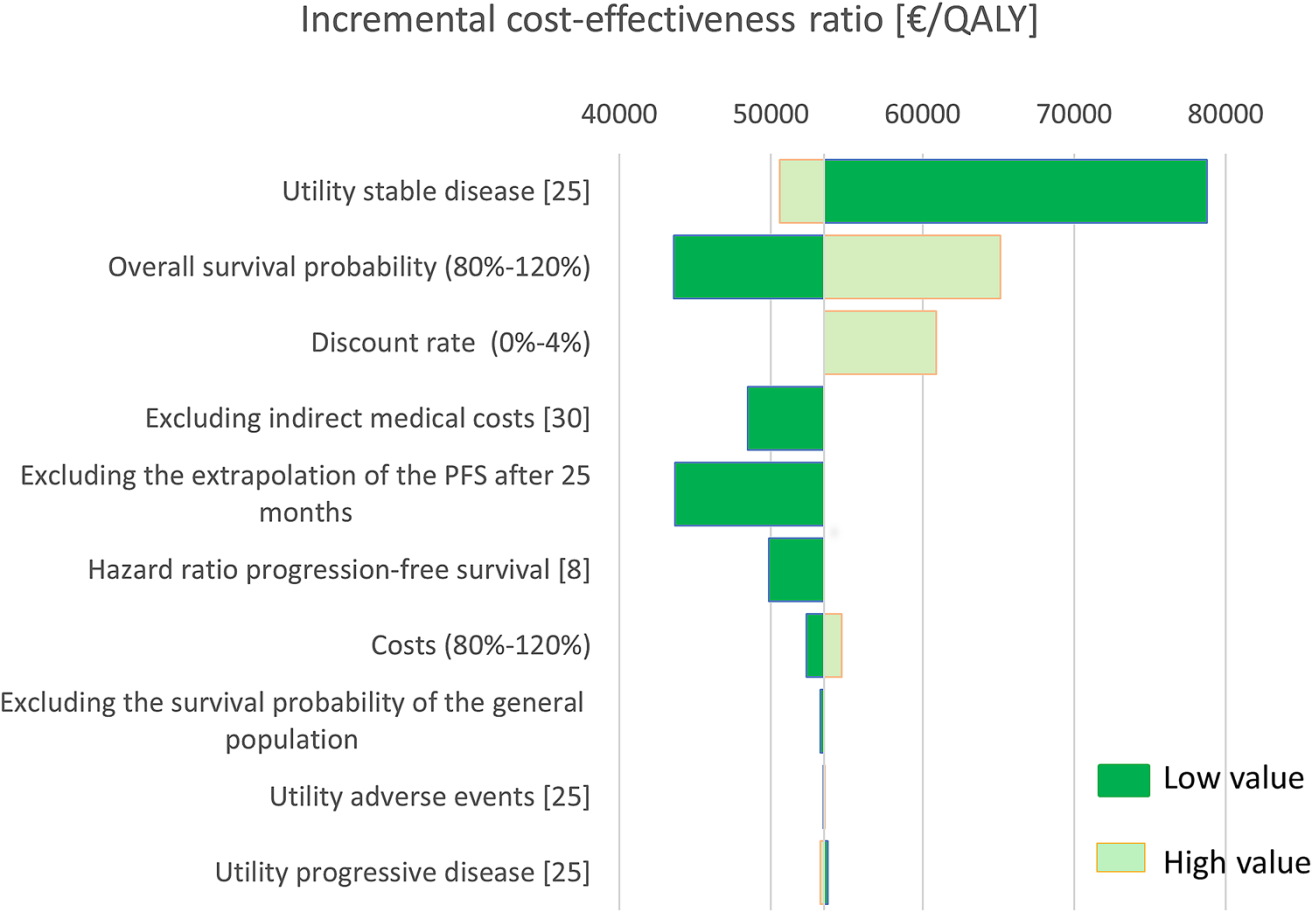
Tornado diagram of the deterministic sensitivity analysis, showing the effects of the upper and lower values of the input parameters on the ICER (base-case at €56,800 per QALY)

The outcomes of the deterministic sensitivity analysis are shown in the Tornado Diagram for the increased price level (Fig. 3). The uncertainty in the utility value of the SD health state has the largest influence on the ICER (range from €50,600 to 78,700). The ICER is not sensitive to changes in the utility in the progressive stage (only limitedly ranging from €53,300 to 53,800) and the adverse events (range from €53,500 to 53,500). The uncertainty in the overall survival probability importantly influences the results (€43,600–65,100). Excluding the extrapolation of the survival from the NETTER-1 study [7] for the full-time horizon and the indirect medical costs shows only a minor impact; it decreases the ICER to a value of €49,900 per QALY and €48,500 per QALY. An equal discount rate of 4% results in an ICER of €60,900 per QALY and no discounting in an ICER of €54,500 per QALY.

## Discussion

In this cost-effectiveness analysis, LO treatment was compared to high-dose O-LAR in The Netherlands. Notably, the focus was on the LO price increase after the acquisition by Novartis [13, 14]. We estimated an ICER of €19,000 per QALY for the initial price and €53,500 per QALY for the increased price; a tripling of the ICER, but still below the WTP threshold. Higher effectiveness of the LO treatment, compared to best supportive care with high-dose O-LAR, is also supported by the findings in earlier studies including various patient sub-groups [9–11, 35–37].

To our knowledge, this is the first economic evaluation executed for LO treatment within the Dutch healthcare system. One cost-effectiveness analysis compared the neuroendocrine tumour treatments Everolimus, Sunitinib, and LO within NICE, the British healthcare market [15], and reported an ICER of £62,168 per QALY comparing LO to best supportive care using the NETTER-1 trail [8] and the utility values from Swinburn and colleagues [25]. We expect that this difference can be mainly explained by additional life-years and QALYs in the control arm, as the NICE study [15] incorporated data from the RADIANT-4 trial [38]. These data indicate that overall survival rates in the control group increase 30 months after randomization [25, 38]; hence, we may underestimate the QALYs gained in the control arm, as we only incorporated the NETTER-1 data with a more limited follow-up.

A limitation of our analysis concerns the assumption that all adverse events occur at the start of treatment—in the absence of detailed data—resulting in absence of appropriate discounting over time. This leads to an overestimation of the total amount of discounted QALYs lost due to adverse events, and therefore, the analysis can be considered as conservative; however, the impact will only be small. Despite that health-related quality-of-life data of the patient population from the NETTER-1 study is published [39], we used the utility values from another source [25], as they provided a clear distinction between the health states used in our model as well as reflecting assessments being population-based. Unfortunately, utility values of some adverse events were unknown [25, 40] and were excluded in the calculation of the mean utility values. We would expect only a small effect on the outcomes of the analyses, since the incidence of most excluded adverse events were not significantly different between the treatment arms. Additionally, the deterministic sensitivity analysis showed that the model is robust for changes in adverse event’s utility values.

Some cost items were not included due to a lack of information [41]; for example, no data were available on productivity losses. This resulted in a primarily healthcare perspective being taken for our evaluation, even though the Dutch guidelines recommend the societal perspective [17, 42]. Yet, we could include indirect non-medical costs in our analysis, with only limited effect on the ICER though. Another limitation in this study concerns the limited set of costs available to populate the PD health state. Besides the standard follow-up outpatient clinic visits, other cost data for this stage are unknown. Yet, since this applies to both the control and the LO group, the impact is expected to be minor. Finally, in this study, only three health states are included (SD, PD, and deceased); however, SD could be subdivided into complete response, partial response, and minimal response. We kept our model straightforward as no utility values were known for these health states separately [25].

Both at the initial and increased price levels, LO is highly likely to be cost-effective at the WTP threshold of €80,000 per QALY. Considering the public scrutiny in relation to the price increase of LO [13, 14] and the partly public funding of LO development [10, 11], an imbalance between this price increase and the investments and risks for the pharmaceutical company has been suggested. The societal debate surrounding LO was primarily related to the balance between public investments in medicine development and drug pricing; do public institutions or governments gain a sufficient return on their investments in research? The absence of comparable effective competition in this case, and for orphan drugs in general, may drive up the price. Currently, the ICER, in relation to the applicable WTP threshold, is the most important outcome to assess the value of new pharmaceutical products [43]. However, this approach has its limitations, as the ICER can be difficult to interpret [44, 45] and does not reflect additional criteria such as affordability and sustainability of health systems or transparency of pricing. Although the assessment of value-based priced products combined with WTP thresholds provides a transparent way to assess the cost-effectiveness of new interventions in healthcare, it should be supplemented by other criteria to align with the public opinion [46–49]. This would especially be important when other pricing strategies fail, e.g., due to an effective monopoly in a specific therapeutic area, as often the case for orphan drugs.

A combined approach, a parallel between traditional cost-effectiveness methods, budget impact analysis, and assessment of medicine pricing, can aid in future decision-making. The ICER can be combined with other values related to affordability, sustainability, access, and transparency. These criteria may include transparency in development costs, including public investments and equity in rewarding all public and private investing partners [50], ensuring early access and budget optimization and the relative cost-effectiveness within a therapeutic area, such as the efficiency frontier approach used in Germany [51, 52]. Public and private investments in research and innovation should remain rewarding for all involved parties, considering benefits in advancing science, enhancing early access of innovations, improving healthcare outcomes, and ensuring the future affordability and sustainability of the healthcare system [53, 54].

## Conclusion

The recent increase in the price of LO treatment resulted in an increase of the ICER from €19,000 per QALY to €53,500 per QALY. As the ICER is below the maximum WTP threshold of €80,000 per QALY, the treatment can be considered cost-effective at the increased price. In general, we suggest that traditional cost-effectiveness methods would benefit from a more extensive approach. Additional criteria to sufficiently capture the full societal value of new medicines, as proposed in this manuscript, should be further researched.

## Supplementary Information

Below is the link to the electronic supplementary material.

Supplementary file1 (PDF 402 KB)

## Data Availability

Data used in the study are available in the mentioned sources. No additional data sources were used.
