# Supplementary material for: Economic evaluation of orphan drug Lutetium-Octreotate vs. Octreotide long-acting release for patients with an advanced midgut neuroendocrine tumour in the Netherlands
Source: Eur J Health Econ. 2021 Apr 7;22(6):991–9. doi: 10.1007/s10198-021-01303-2 (PMC8275500; doi:10.1007/s10198-021-01303-2)
Supplement: Supplementary file 1 — Supplementary file1 (PDF 402 KB) [file 10198_2021_1303_MOESM1_ESM.pdf]

Supplementary appendix

Economic evaluation of orphan drug Lutetium-Octreotate vs. Octreotide long-acting release for patients with an advanced Midgut Neuroendocrine Tumour in the Netherlands

Contents

Supplementary appendix 1 – Overview costs……………………….…………………………2

Supplementary appendix 2 – Calculation of burden of disease………………………………..3

Supplementary appendix 3 - Fits of modelled survival curves………………………...……4

Supplementary appendix 4 - Cost-effectiveness plane of the initial and increased price levels of the LO treatment compared to best usual care…………………………………………....5

References……………………………………………………………………………………...6

Supplementary appendix 1 – Overview costs

Actions per group	Costs per cycle per capita (€)	Source
**LO + O-LAR**		
**Medicine costs**	20,743.- (Increased price)	[1]
	4,000.- (Initial price)	[2]
**Amino Acids Infusion**	89.38	[3]
**Granisteron 3mg**	62.13	[3]
**Hotel costs (per night)**	685.78	[4]
**MRI**	473.82	[4]
**CT-scan**	304.14	[4]
**Adverse events control**	80,920.-	[5]
**Injection O-LAR 30 mg**	537.25	[3]
**General practitioner visit**	35.22	[4]
**High dose O-LAR**		
**Injection O-LAR 60 mg**	1,074.50	[3]
**General practitioner visit**	35.22	[4]
**CT-scan**	304.14	[4]
**Visit outpatient clinic**	180.85	[4]
**Follow up both groups**		
**Visit outpatient clinic**	180.85	[4]
**Blood tests**	11.77	[4]
**Indirect medical costs**		
**Indirect medical costs (other-month)**	431.64*	[6]
**End-of-Life (last-month)**	46,720.56*	[6]

*LO = Lutetium-Octreotate. O-LAR = Octreotide long-acting release. *Age-dependent, costs in first cycle given.*

Supplementary appendix 2 – Calculation of the burden of disease

Stage	Utility (SD) [7]	OS (range) [years] [8]	QALY (Utility*OS)	SD around PsA (SD*OS)	Likelihood of applicable threshold being €80.000 [%] [9]
**Progressive**	0.612 (0.24)	3.42 (2.25-5.50)	2.09 (1.38-3.37)	0.82 (0.54-1.32)	100 (91-100)
**Stable**	0.771 (0.20)	3.42 (2.25-5.50)	2.63 (1.73-4.24)	0.68 (0.45-1.10)	99.9 (80.2-100)

<u>Supplementary appendix 3 - Fits of modelled survival curves</u>

Progression-free survival:

Preferred distribution: **e**xponential distribution

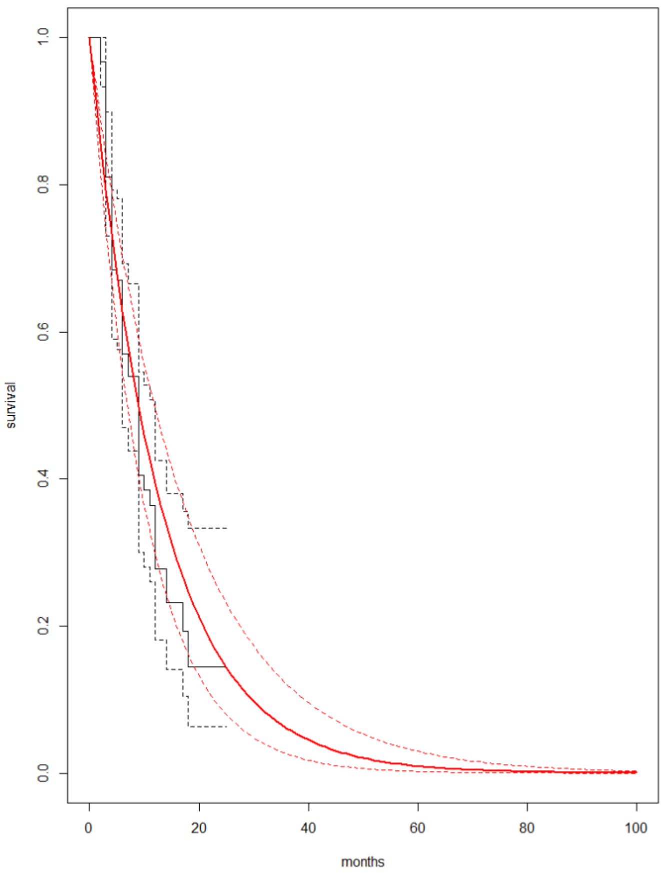

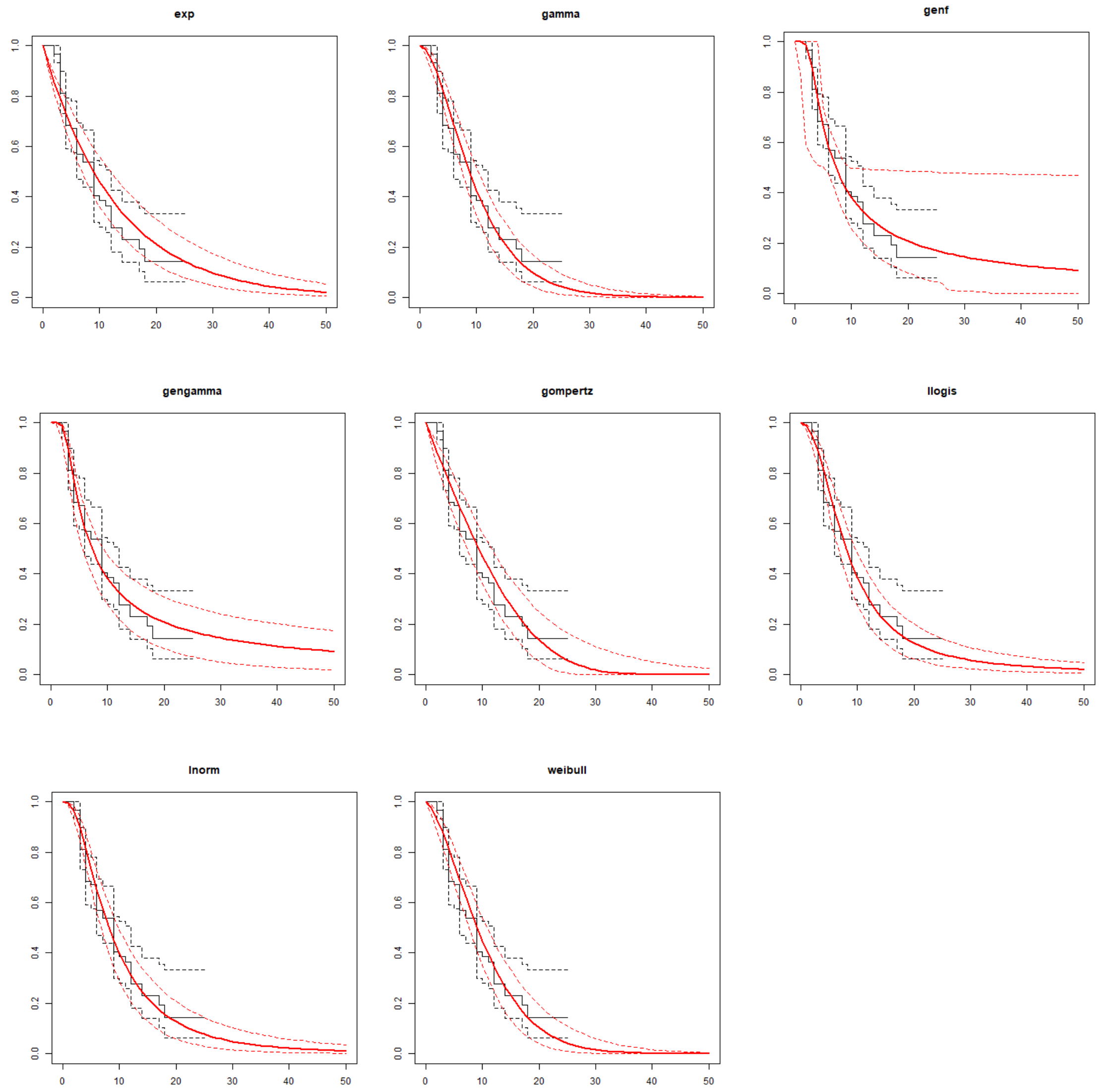

Overall survival:

preferred distribution: gamma distribution

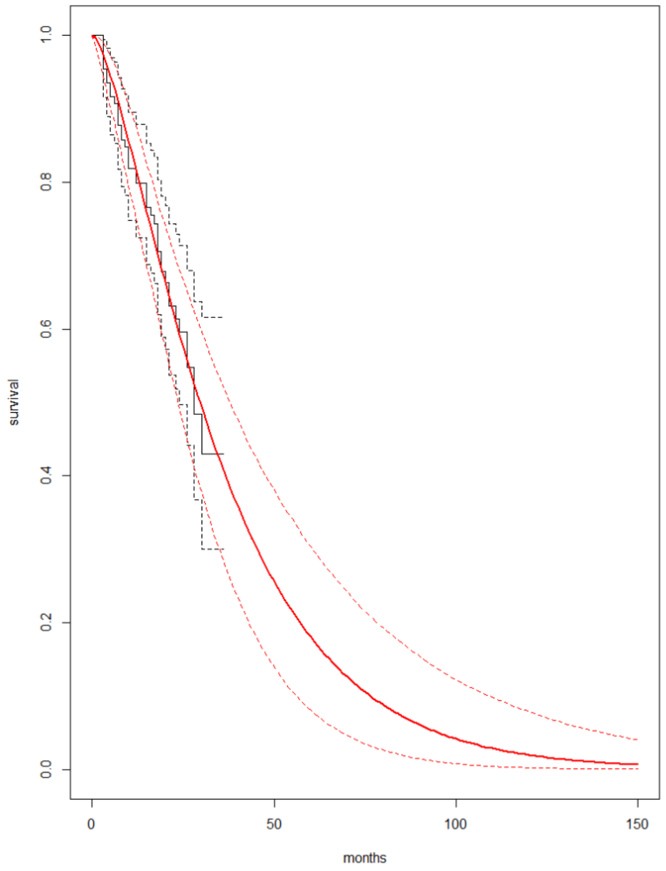

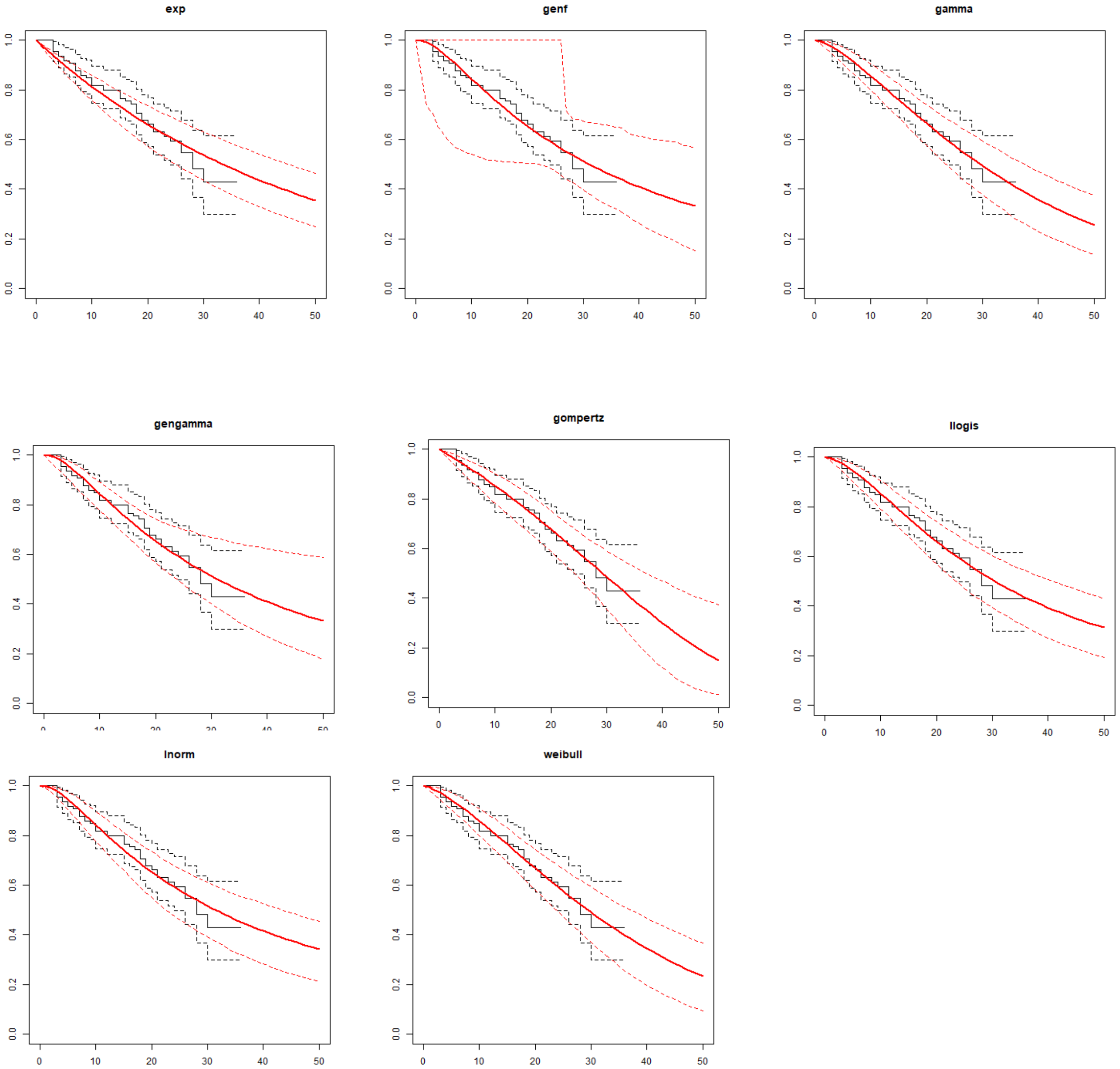

Supplementary appendix 4 - Cost-effectiveness plane of the initial and increased price levels of the LO treatment compared to best usual care

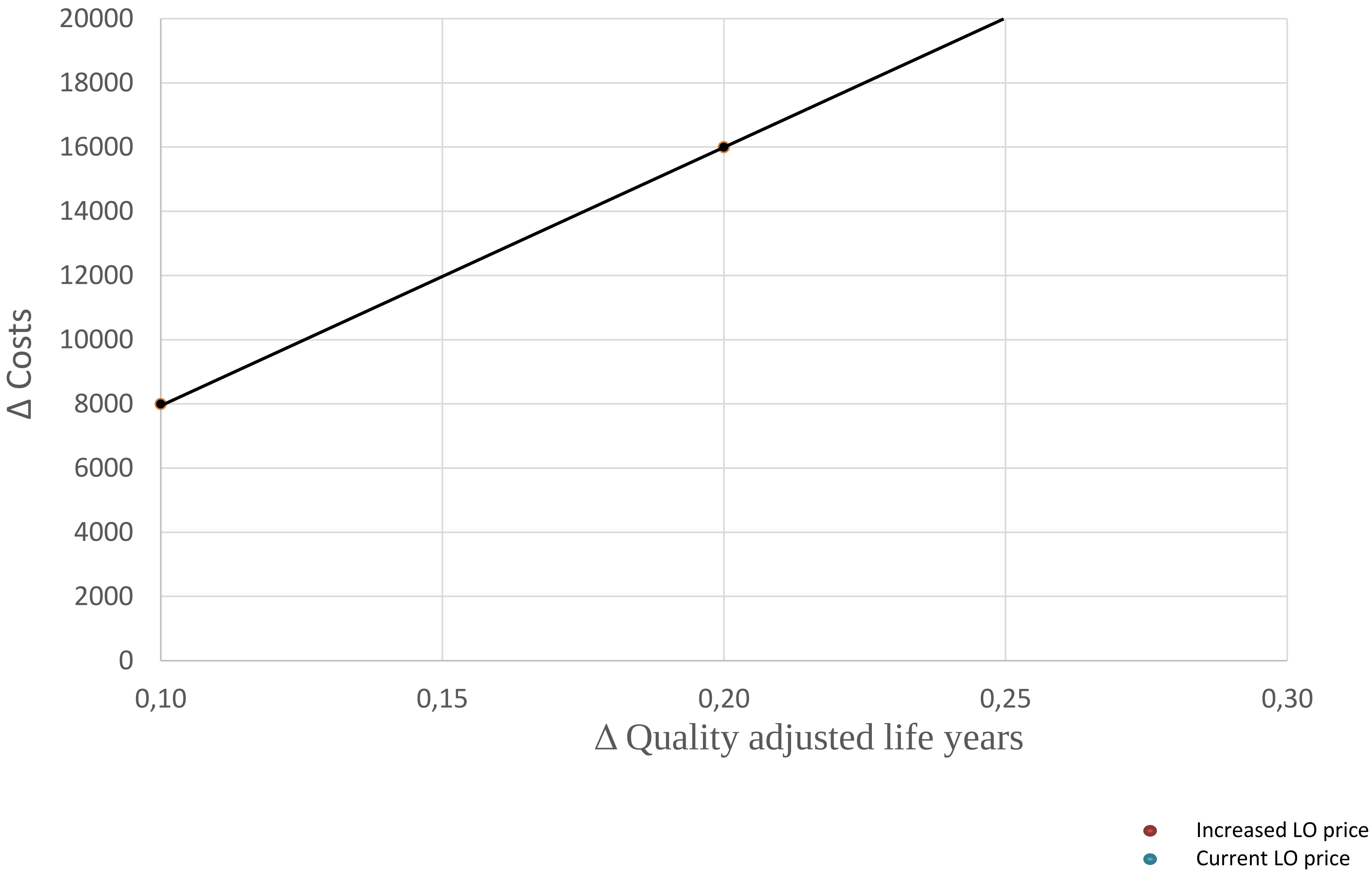

References

1. Nederlandse zorgautoriteit, Prestatie- en tariefbeschikking Add-ongeneesmiddelen, juli 2018 (op aanvraag) – TB/REG-18673-01: https://puc.overheid.nl/nza/doc/PUC_242394_22/1/ [Accessed June 07, 2020]

2. Hordijk L. Reconstructie Lutetium-Octreotaat. Nederlands Tijdschrift Geneeskunde 2019; 163: D3744

3. Zorginstituut Nederland URL: https://medicijnkosten.nl [Accessed May 15, 2019]

4. Hakkaart-van Roijen L, Van der Linden N, Bouwmans C, Kanters T, Tan SS. Kostenhandleiding. Methodologie van kostenonderzoek en referentieprijzen voor economische evaluaties in de gezondheidszorg. In opdracht van Zorginstituut Nederland. Geactualiseerde versie. 2015.

5. Strosberg JR, El-Haddad G, Wolin E, et al. Phase 3 trial of ^{177}Lu-DOTATATE for midgut neuroendocrine tumors. New England Journal of Medicine 2017; 376(2): 125-135.

6. van Baal PH, Wong A, Slobbe LC, Polder JJ, Brouwer WB, de Wit GA. Standardizing the inclusion of indirect medical costs in economic evaluations. Pharmacoeconomics 2011; 29(3): 175-187.

7. Swinburn P, Wang J, Chandiwana D, Mansoor W, & Lloyd A. Elicitation of health state utilities in neuroendocrine tumours. Journal of medical economics 2012; 15(4): 681-687.

8. Man D, Jingjing W, Zhan S, Xiaoyi Z. Prognosis of patients with neuroendocrine tumor: a SEER database analysis. Cancer management and research 2018; 10: 5629.

9. iDBC – iMTA Disease Burden Calculator. URL: https://imta.shinyapps.io/iDBC/
